# Supplementary material for: Time-series transcriptome analysis identified differentially expressed genes in broiler chicken infected with mixed Eimeria species
Source: Front Genet. 2022 Aug 8;13:886781. doi: 10.3389/fgene.2022.886781 (PMC9393255; doi:10.3389/fgene.2022.886781)
Supplement: Supplementary file 2 [file DataSheet1.ZIP › 7dpi_GO.Gsea.1625106495923/GOMF_STRUCTURAL_CONSTITUENT_OF_RIBOSOME.html]

Details for gene set GOMF\_STRUCTURAL\_CONSTITUENT\_OF\_RIBOSOME[GSEA]

|  || Dataset | TMM\_7dpi\_gct\_format\_7dpi\_gct\_format.Class\_4dpi.cls #PC\_versus\_NC.Class\_4dpi.cls #PC\_versus\_NC\_repos |
| Phenotype | Class\_4dpi.cls#PC\_versus\_NC\_repos |
| Upregulated in class | 1 |
| GeneSet | GOMF\_STRUCTURAL\_CONSTITUENT\_OF\_RIBOSOME |
| Enrichment Score (ES) | 0.6321381 |
| Normalized Enrichment Score (NES) | 3.303854 |
| Nominal p-value | 0.0 |
| FDR q-value | 0.0 |
| FWER p-Value | 0.0 |
Table: GSEA Results Summary

  

Fig 1: Enrichment plot: GOMF\_STRUCTURAL\_CONSTITUENT\_OF\_RIBOSOME      
 Profile of the Running ES Score & Positions of GeneSet Members on the Rank Ordered List

  

| SYMBOL | TITLE | RANK IN GENE LIST | RANK METRIC SCORE | RUNNING ES | CORE ENRICHMENT || 1 | MRPS12 | na | 242 | 0.999 | 0.0075 | Yes |
| 2 | MRPS6 | na | 315 | 0.928 | 0.0274 | Yes |
| 3 | MRPL30 | na | 351 | 0.883 | 0.0491 | Yes |
| 4 | NDUFA7 | na | 371 | 0.857 | 0.0714 | Yes |
| 5 | MRPL43 | na | 504 | 0.741 | 0.0810 | Yes |
| 6 | RPS28 | na | 529 | 0.721 | 0.0991 | Yes |
| 7 | MRPL23 | na | 564 | 0.704 | 0.1159 | Yes |
| 8 | MRPL12 | na | 579 | 0.694 | 0.1341 | Yes |
| 9 | MRPL57 | na | 643 | 0.665 | 0.1474 | Yes |
| 10 | RPS29 | na | 740 | 0.617 | 0.1566 | Yes |
| 11 | MRPL54 | na | 779 | 0.596 | 0.1700 | Yes |
| 12 | MRPS33 | na | 798 | 0.591 | 0.1850 | Yes |
| 13 | RPS27L | na | 833 | 0.579 | 0.1983 | Yes |
| 14 | MRPL35 | na | 853 | 0.571 | 0.2127 | Yes |
| 15 | MRPL10 | na | 872 | 0.563 | 0.2269 | Yes |
| 16 | MRPL55 | na | 880 | 0.561 | 0.2420 | Yes |
| 17 | MRPS16 | na | 950 | 0.538 | 0.2512 | Yes |
| 18 | RPL26L1 | na | 1025 | 0.512 | 0.2592 | Yes |
| 19 | MRPL21 | na | 1141 | 0.472 | 0.2628 | Yes |
| 20 | MRPL15 | na | 1217 | 0.450 | 0.2690 | Yes |
| 21 | MRPS23 | na | 1344 | 0.420 | 0.2701 | Yes |
| 22 | UBA52 | na | 1369 | 0.413 | 0.2796 | Yes |
| 23 | MRPL46 | na | 1380 | 0.409 | 0.2902 | Yes |
| 24 | MRPL28 | na | 1382 | 0.409 | 0.3016 | Yes |
| 25 | RPS27 | na | 1424 | 0.399 | 0.3093 | Yes |
| 26 | MRPL9 | na | 1437 | 0.396 | 0.3193 | Yes |
| 27 | MRPL3 | na | 1475 | 0.387 | 0.3270 | Yes |
| 28 | MRPL42 | na | 1521 | 0.375 | 0.3337 | Yes |
| 29 | MRPL19 | na | 1535 | 0.370 | 0.3429 | Yes |
| 30 | RPS19 | na | 1540 | 0.368 | 0.3529 | Yes |
| 31 | MRPL47 | na | 1550 | 0.367 | 0.3623 | Yes |
| 32 | RPL38 | na | 1576 | 0.361 | 0.3703 | Yes |
| 33 | RPL36 | na | 1577 | 0.361 | 0.3804 | Yes |
| 34 | MRPL1 | na | 1639 | 0.348 | 0.3850 | Yes |
| 35 | MRPS17 | na | 1651 | 0.345 | 0.3937 | Yes |
| 36 | MRPL34 | na | 1672 | 0.339 | 0.4015 | Yes |
| 37 | MRPS35 | na | 1686 | 0.336 | 0.4097 | Yes |
| 38 | MRPS11 | na | 1692 | 0.335 | 0.4187 | Yes |
| 39 | MRPL33 | na | 1696 | 0.333 | 0.4277 | Yes |
| 40 | MRPS18C | na | 1729 | 0.325 | 0.4341 | Yes |
| 41 | MRPL37 | na | 1736 | 0.324 | 0.4427 | Yes |
| 42 | RPL23A | na | 1779 | 0.313 | 0.4479 | Yes |
| 43 | RPL29 | na | 1780 | 0.313 | 0.4566 | Yes |
| 44 | RPS15 | na | 1804 | 0.306 | 0.4632 | Yes |
| 45 | RPLP1 | na | 1813 | 0.304 | 0.4710 | Yes |
| 46 | RPL36A | na | 1833 | 0.301 | 0.4778 | Yes |
| 47 | RPL19 | na | 1834 | 0.300 | 0.4862 | Yes |
| 48 | MRPL2 | na | 1843 | 0.299 | 0.4939 | Yes |
| 49 | MRPS21 | na | 1864 | 0.296 | 0.5004 | Yes |
| 50 | MRPL16 | na | 1884 | 0.291 | 0.5070 | Yes |
| 51 | RPL39L | na | 1966 | 0.277 | 0.5079 | Yes |
| 52 | RPL37A | na | 1983 | 0.274 | 0.5142 | Yes |
| 53 | MRPL41 | na | 2082 | 0.257 | 0.5131 | Yes |
| 54 | MRPL24 | na | 2093 | 0.254 | 0.5194 | Yes |
| 55 | MRPL13 | na | 2109 | 0.251 | 0.5251 | Yes |
| 56 | MRPS9 | na | 2193 | 0.238 | 0.5248 | Yes |
| 57 | RPL22 | na | 2201 | 0.237 | 0.5308 | Yes |
| 58 | RPL35A | na | 2241 | 0.231 | 0.5340 | Yes |
| 59 | RSL24D1 | na | 2281 | 0.225 | 0.5370 | Yes |
| 60 | MRPL14 | na | 2284 | 0.224 | 0.5431 | Yes |
| 61 | RPL34 | na | 2286 | 0.224 | 0.5492 | Yes |
| 62 | RPL27 | na | 2300 | 0.222 | 0.5543 | Yes |
| 63 | MRPS14 | na | 2305 | 0.222 | 0.5602 | Yes |
| 64 | RPS26 | na | 2332 | 0.216 | 0.5640 | Yes |
| 65 | RPL14 | na | 2343 | 0.215 | 0.5692 | Yes |
| 66 | RPS24 | na | 2347 | 0.214 | 0.5749 | Yes |
| 67 | MRPS18A | na | 2363 | 0.213 | 0.5796 | Yes |
| 68 | RPL23 | na | 2383 | 0.209 | 0.5839 | Yes |
| 69 | RPL7L1 | na | 2403 | 0.207 | 0.5880 | Yes |
| 70 | RPS7 | na | 2413 | 0.204 | 0.5930 | Yes |
| 71 | MRPS34 | na | 2496 | 0.193 | 0.5915 | Yes |
| 72 | MRPL18 | na | 2512 | 0.189 | 0.5955 | Yes |
| 73 | MRPS5 | na | 2649 | 0.170 | 0.5888 | Yes |
| 74 | MRPS25 | na | 2661 | 0.169 | 0.5926 | Yes |
| 75 | RPL7 | na | 2670 | 0.168 | 0.5966 | Yes |
| 76 | RPL31 | na | 2693 | 0.164 | 0.5993 | Yes |
| 77 | RPL24 | na | 2734 | 0.160 | 0.6004 | Yes |
| 78 | MRPL22 | na | 2747 | 0.158 | 0.6038 | Yes |
| 79 | RPS8 | na | 2771 | 0.154 | 0.6062 | Yes |
| 80 | MRPL20 | na | 2783 | 0.153 | 0.6095 | Yes |
| 81 | RPL21 | na | 2810 | 0.149 | 0.6115 | Yes |
| 82 | RPL32 | na | 2870 | 0.142 | 0.6105 | Yes |
| 83 | RPL13 | na | 2902 | 0.138 | 0.6118 | Yes |
| 84 | RPS17 | na | 2988 | 0.128 | 0.6082 | Yes |
| 85 | RPS15A | na | 3019 | 0.124 | 0.6091 | Yes |
| 86 | RPS13 | na | 3028 | 0.122 | 0.6119 | Yes |
| 87 | DAP3 | na | 3039 | 0.121 | 0.6144 | Yes |
| 88 | RPLP2 | na | 3040 | 0.121 | 0.6178 | Yes |
| 89 | RPL6 | na | 3048 | 0.121 | 0.6205 | Yes |
| 90 | RPS14 | na | 3050 | 0.120 | 0.6238 | Yes |
| 91 | RPL27A | na | 3078 | 0.116 | 0.6248 | Yes |
| 92 | RPS20 | na | 3085 | 0.115 | 0.6275 | Yes |
| 93 | SRBD1 | na | 3139 | 0.108 | 0.6260 | Yes |
| 94 | RPL9 | na | 3146 | 0.107 | 0.6285 | Yes |
| 95 | MRPL27 | na | 3152 | 0.107 | 0.6311 | Yes |
| 96 | MRPS7 | na | 3210 | 0.100 | 0.6291 | Yes |
| 97 | MRPL51 | na | 3225 | 0.098 | 0.6307 | Yes |
| 98 | MRPS22 | na | 3240 | 0.095 | 0.6321 | Yes |
| 99 | RPS25 | na | 3324 | 0.084 | 0.6275 | No |
| 100 | RPL30 | na | 3365 | 0.078 | 0.6263 | No |
| 101 | RPS10 | na | 3396 | 0.074 | 0.6259 | No |
| 102 | RPL11 | na | 3414 | 0.072 | 0.6264 | No |
| 103 | MRPS31 | na | 3424 | 0.071 | 0.6277 | No |
| 104 | RPL7A | na | 3462 | 0.067 | 0.6264 | No |
| 105 | RPL35 | na | 3487 | 0.064 | 0.6262 | No |
| 106 | RPS21 | na | 3592 | 0.053 | 0.6189 | No |
| 107 | RPL22L1 | na | 3609 | 0.051 | 0.6190 | No |
| 108 | RPS16 | na | 3626 | 0.051 | 0.6191 | No |
| 109 | RPL18A | na | 3668 | 0.045 | 0.6169 | No |
| 110 | RPS11 | na | 3727 | 0.037 | 0.6130 | No |
| 111 | RPS2 | na | 3817 | 0.025 | 0.6063 | No |
| 112 | MRPL32 | na | 3822 | 0.025 | 0.6066 | No |
| 113 | RPL10A | na | 3828 | 0.024 | 0.6069 | No |
| 114 | RPL3 | na | 3883 | 0.018 | 0.6028 | No |
| 115 | RPL37 | na | 4179 | -0.015 | 0.5784 | No |
| 116 | RPS27A | na | 4189 | -0.016 | 0.5781 | No |
| 117 | RPL8 | na | 4274 | -0.027 | 0.5718 | No |
| 118 | RPS12 | na | 4369 | -0.037 | 0.5649 | No |
| 119 | RPS3A | na | 4499 | -0.050 | 0.5554 | No |
| 120 | RPL5 | na | 4509 | -0.051 | 0.5561 | No |
| 121 | MRPS2 | na | 4677 | -0.068 | 0.5439 | No |
| 122 | RPL15 | na | 4832 | -0.084 | 0.5333 | No |
| 123 | RPL12 | na | 4912 | -0.094 | 0.5293 | No |
| 124 | RPL4 | na | 5112 | -0.114 | 0.5157 | No |
| 125 | RPLP0 | na | 5368 | -0.137 | 0.4981 | No |
| 126 | RPS4Y1 | na | 5409 | -0.140 | 0.4986 | No |
| 127 | RPS3 | na | 5595 | -0.158 | 0.4874 | No |
| 128 | MRPS36 | na | 5609 | -0.160 | 0.4908 | No |
| 129 | MRPL17 | na | 5768 | -0.177 | 0.4824 | No |
| 130 | RPL17 | na | 7417 | -0.353 | 0.3536 | No |
| 131 | RPS23 | na | 7461 | -0.357 | 0.3599 | No |
| 132 | RPS6 | na | 7644 | -0.376 | 0.3551 | No |
| 133 | MRPS30 | na | 8183 | -0.444 | 0.3222 | No |
Table: GSEA details [plain text format]

  

Fig 2: GOMF\_STRUCTURAL\_CONSTITUENT\_OF\_RIBOSOME      
 Blue-Pink O' Gram in the Space of the Analyzed GeneSet

  

Fig 3: GOMF\_STRUCTURAL\_CONSTITUENT\_OF\_RIBOSOME: Random ES distribution      
 Gene set null distribution of ES for **GOMF\_STRUCTURAL\_CONSTITUENT\_OF\_RIBOSOME**
